# Resurrection of an ancient inflammatory locus reveals switch to caspase-1 specificity on a caspase-4 scaffold

**DOI:** 10.1016/j.jbc.2022.101931

**Published:** 2022-04-12

**Authors:** Betsaida Bibo-Verdugo, Isha Joglekar, Mithun N. Karadi Giridhar, Monica L. Ramirez, Scott J. Snipas, A. Clay Clark, Marcin Poreba, Guy S. Salvesen

**Affiliations:** 1 Sanford Burnham Prebys Medical Discovery Institute, La Jolla, California, USA; 2 Department of Biology, University of Texas at Arlington, Arlington, Texas, USA; 3 Department of Pharmacology, University of California San Diego, La Jolla, California, USA; 4 Department of Bioorganic Chemistry, Wroclaw University of Science and Technology, Wroclaw, Poland

**Keywords:** caspase, cell death, inflammation, interleukin 1, protein evolution, ACC, 7-amino-4-carbamoylmethylcoumarin, AFC, 7-amino-4-trifluoromethylcoumarin, ASR, ancestral sequence reconstruction, CARD, caspase activation and recruitment domain, DAMP, damage-associated molecular pattern, IL-1β, interleukin-1β, IL-18, interleukin-18, pro-IL-1β, inactive precursor of IL-1β, pro-IL-18, inactive precursor of IL-18

## Abstract

Pyroptosis is a mechanism of inflammatory cell death mediated by the activation of the prolytic protein gasdermin D by caspase-1, caspase-4, and caspase-5 in human, and caspase-1 and caspase-11 in mouse. In addition, caspase-1 amplifies inflammation by proteolytic activation of cytokine interleukin-1β (IL-1β). Modern mammals of the order Carnivora lack the caspase-1 catalytic domain but express an unusual version of caspase-4 that can activate both gasdermin D and IL-1β. Seeking to understand the evolutionary origin of this caspase, we utilized the large amount of data available in public databases to perform ancestral sequence reconstruction of an inflammatory caspase of a Carnivora ancestor. We expressed the catalytic domain of this putative ancestor in *Escherichia coli*, purified it, and compared its substrate specificity on synthetic and protein substrates to extant caspases. We demonstrated that it activates gasdermin D but has reduced ability to activate IL-1β. Our reconstruction suggests that caspase-1 was lost in a Carnivora ancestor, perhaps upon a selective pressure for which the generation of biologically active IL-1β by caspase-1 was detrimental. We speculate that later, a Carnivora encountered selective pressures that required the production of IL-1β, and caspase-4 subsequently gained this activity. This hypothesis would explain why extant Carnivora possess an inflammatory caspase with caspase-1 catalytic function placed on a caspase-4 scaffold.

The cell death mechanisms of apoptosis and pyroptosis are executed by proteases of the caspase family (1). Caspases’ distinctive cleavage specificity of signature substrate proteins results in programmed cell death of distinct phenotypes. For example, apoptosis is noninflammatory (2, 3). The cell conformation changes as it blebs, shrinks, and decays without interrupting its immediate environment. In contrast, pyroptosis displays a lytic phenotype and is highly inflammatory (4). This biochemical process triggers the response of the innate immune system and leads to the clearance of pathogens (5).

Inflammatory caspases drive two recognized pyroptotic pathways. Caspase-1 activates the canonical pathway (6), whereas mouse caspase-4 (also known as caspase-11) and human caspase-4 and caspase-5 initiate the noncanonical pathway (7, 8). The field is often confusing about the relationship between caspase-4 and caspase-11, which are usually considered to be orthologs, even though they may have different biological activities (9). Accordingly, we used caspase-11 exclusively for the mouse protein and caspase-4 for the human. Pyroptosis execution depends on cleavage of the effector protein, gasdermin D (10, 11). Cleavage of gasdermin D by inflammatory caspases results in the liberation of the lytic N-terminal domain (12). The free N-terminal domain oligomerizes and initiates the lytic phenotype by contributing to pore formation at the plasma membrane (12, 13). Gasdermin D is known to allow the release of damage-associated molecular patterns (DAMPs) and inflammatory cytokines interleukin-1β (IL-1β) and interleukin-18 (IL-18) (14, 15, 16, 17).

The inactive precursors of IL-1β and IL-18 (pro-IL-1β and pro-IL-18) become proteolytically activated by removal of their propeptide (18, 19, 20, 21). Biochemical studies and genetic animal models suggest that conversion of pro-IL-1β is a function performed mainly by caspase-1 in human and mouse (7, 9, 22). Mouse caspase-11, and human caspase-4 and caspase-5, have inadequate capacity to cleave pro-IL-1β (9, 22). Cleavage of pro-IL-18 is less restricted as it can be cleaved by human caspase-1 and caspase-4 with similar efficiency (9). Caspase-1 is activated downstream of caspase-11 in an NLRP3 (NOD-, LRR-, and pyrin domain–containing protein 3) inflammasome–dependent manner, providing an alternative mechanism to produce IL-1β during noncanonical pyroptosis (7, 23). Hence, pyroptosis is an essential first line of defense against pathogens by eliminating their replicating niche and influences downstream immune responses by producing inflammatory IL-1β and IL-18 (24, 25). The proteolytic efficiency differences among caspases of the canonical and noncanonical pathways on pro-IL-1β highlight a pivotal difference in their contribution to innate immunity.

Inflammatory caspases consist of a caspase activation and recruitment domain (CARD) followed by a catalytic domain with proteolytic function (26). Typically, *CASP1* and *CASP4* are contiguous genes, except in primates where *CASP5* lies between these two genes (27). The dog genome contains a single caspase-1 and caspase-4-like gene (Fig. 1*A*). This gene encodes a protein called “hybrid caspase-1/caspase-4” or “hybrid inflammatory caspase,” (28) which contains a caspase-1 CARD followed by a caspase-4 CARD and a catalytic domain (Fig. 1*A*). Early studies suggest that this gene originated *via* the deletion of a chromosomal segment encoding the caspase-1 catalytic domain and exon 1 of *CASP4* (28).

**Figure 1:**
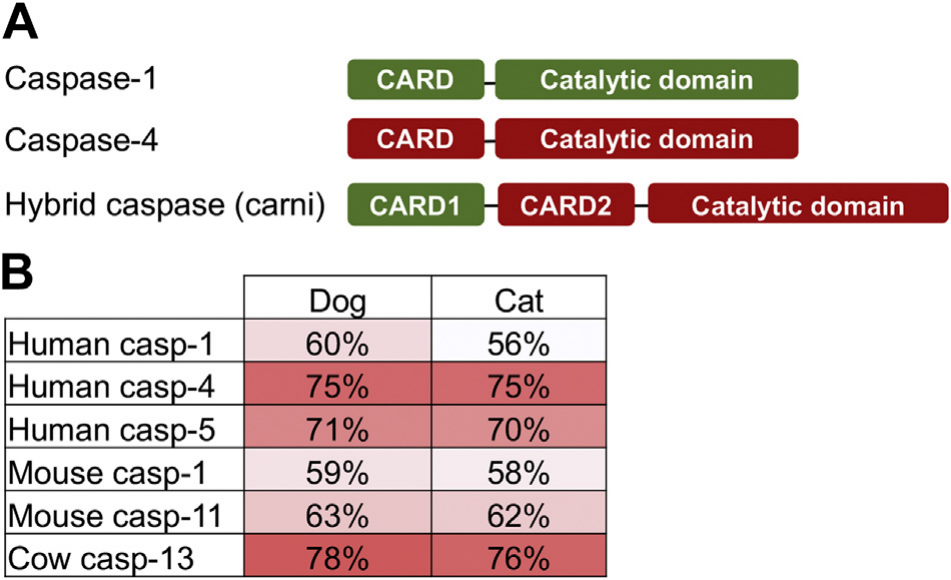
**The protein domains of inflammatory caspases.***A*, an inflammatory caspase found in the order Carnivora contains two consecutive CARDs and one catalytic domain. *B*, the percent identity of inflammatory caspase catalytic domain sequences of two Carnivora (dog and cat) to other mammals demonstrates that they are more closely related to caspase-4 than to caspase-1. Cow caspase-13 is the most probable ortholog of human caspase-4. CARD, caspase activation and recruitment domain.

In our previous report of inflammatory cell death in mammals of the order Carnivora, we observed that the catalytic domain of the dog inflammatory caspase behaved like caspase-1 catalytically, despite its sequence identity to caspase-4 (29). Moreover, the caspase-1 and caspase-4-like gene seems to be common in mammals of this order (29, 30). Given the importance of caspase-1 in inflammation through pyroptosis signaling and pro-IL-1β processing, we hypothesized that the loss of caspase-1 catalytic domain in Carnivora became compensated by reshaping the caspase-4 catalytic domain and function. We looked for caspases with similar domain architecture in mammals to test this hypothesis and approximate how the caspase-1 and caspase-4-like gene originated. Previous studies suggest that the inflammatory caspase locus is under extreme selective pressure, leading for instance toward a new inflammatory caspase in higher primates (27, 31). To understand the evolution of this locus in Carnivora, we set out to reconstruct the most probable protein sequence that represents the ancestor from whom the inflammatory caspase catalytic domain diverged in these mammals. Finally, we evaluated the substrate cleavage and specificity of this hypothetical ancestral inflammatory caspase.

## Results

### Sequence-based classification of the Carnivora inflammatory caspase

To investigate the occurrence of inflammatory caspases with the CARD–CARD–catalytic domain architecture in mammals, we performed a BLAST analysis search using the dog inflammatory caspase sequence as a query (UniProt: A9YEF4). We found a total of 24 proteins (Table S1), 23 of which were found in mammals of the order Carnivora (of the more than 270 reported species of Carnivora), and only one belonging to the order Artiodactyla, namely sheep. The high prevalence of the hybrid inflammatory caspase in Carnivora and the lack of a caspase-1 ortholog in this clade suggest that *CASP1* deletion occurred early in the evolution of these mammals.

Cladograms revealed that the first CARD in the hybrid inflammatory caspase grouped with caspase-1, whereas the subsequent CARD grouped with caspase-4 CARDs (Fig. S1). In addition, the catalytic domain grouped with caspase-4 (Figs. 1*B* and S1). Consequently, we hypothesize that, regardless of the CARD-dependent activation mechanism, the catalytic activity and specificity of the catalytic domain in the Carnivora inflammatory caspases would be caspase-4 like.

### Dog inflammatory caspase shows caspase-1-like specificity

Human caspase-4 and mouse caspase-11 cleave gasdermin D more efficiently than they cleave pro-IL-1β (9, 22). Accordingly, our hypothesis predicts that the catalytic domain of the Carnivora inflammatory caspase would cleave gasdermin D better than pro-IL-1β. We recombinantly expressed and purified the CARD-depleted version of the dog inflammatory caspase. We obtained an active and processed protein consisting of a large (p20) and a small (p10) subunit (Fig. S2).

**Figure 2:**
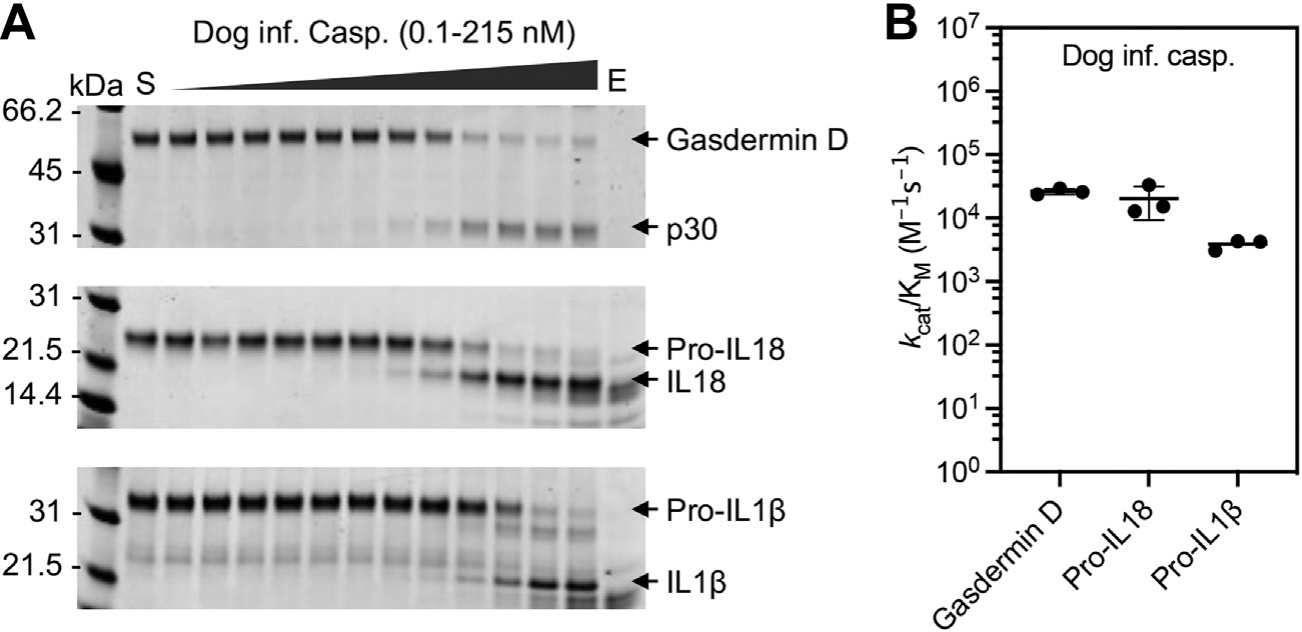
**The dog inflammatory caspase cleaves the key pyroptosis proteins gasdermin D, pro-IL-18, and pro-IL-1β.***A*, recombinant protein substrates—4 μM mouse gasdermin D, pro-IL-18, and pro-IL-1β—were cleaved *in vitro* with a dilution series of the dog inflammatory caspase and analyzed by Coomassie blue–stained SDS-PAGE. S and E represent the substrate- and enzyme-only controls. *B*, the catalytic parameter, *k*_cat_/K_m_, of the dog inflammatory caspase against protein substrates was calculated based on this SDS-PAGE analysis. The figure shows average and SD of three experiments. Pro-IL-1β, inactive precursor of interleukin-1β; pro-IL-18, inactive precursor of interleukin-18.

Utilizing *in vitro* cleavage assays, we calculated the catalytic parameter *k*_cat_/K_m_ of the dog inflammatory caspase toward recombinant protein substrates gasdermin D, pro-IL-18, and pro-IL-1β. Strikingly, the dog inflammatory caspase cleaved gasdermin D, pro-IL-18, and pro-IL-1β with similar efficiency to mouse and human caspase-1 (9, 22) (Fig. 2). We conclude that the dog inflammatory caspase is endowed with the biochemical properties that allow it to perform caspase-1 functions.

Caspase specificity is dictated by interactions with amino acids in a bipartite sequence motif surrounding the cleaved peptide bond. Most caspases, including caspase-1, recognize the N-terminal portion of this motif. In contrast, caspase-4 recognizes the C-terminal side (9). Because the usage of substrate recognition subsites is an elemental distinction between caspase-1 and caspase-4, we tested the specificity of the dog inflammatory caspase on peptides corresponding to the recognition motif by using a set of internally quenched fluorogenic substrates. Like caspase-1 (9), the dog inflammatory caspase was more influenced by the N-terminal region than by the C-terminal region (Table 1 and Fig. S3).

**Table 1:** *k*_cat_/*K*_*m*_ of the dog inflammatory caspase for internally quenched substrates surrounding the gasdermin D cleavage site motif sequence

Substrate sequence	Dog inflammatory caspase			
	*k*_cat_ (s^−1^)	*K*_*m*_ (μM)	10^3^ × *k*_cat_/*K*_*m*_ (s^−1^ M^−1^)	*k*_cat_/*K*_*m*_ (fold change)
SLLSDG	1.06 ± 0.3	87 ± 16	12.0 ± 1.3	1
SLLSDGI	3.75 ± 0.7	52 ± 16	74.4 ± 11	6
SLLSDGID	2.23 ± 1	49 ± 6.8	44.5 ± 14	4
SLLSDGIDE	3.00 ± 0.5	38 ± 8	79.6 ± 4.9	7
SLLSDGIDEE	2.72 ± 0.007	47 ± 4	58.4 ± 5.1	5
SDGIDEE	0.02 ± 0	38 ± 0	0.77 ± 0	1
LSDGIDE	0.30 ± 0.05	115 ± 36	2.6 ± 0.42	3
LLSDGID	4.93 ± 1.5	22 ± 8	222.6 ± 13	288

The peptides are flanked by an N-terminal fluorophore and a C-terminal quencher as described in the Experimental procedures section. Cleavage occurs after the underlined Asp residue. Fold change in *k*_cat_/*K*_*m*_ was calculated relative to the lowest value within each group. Data show average and SD of two independent experiments.

**Figure 3:**
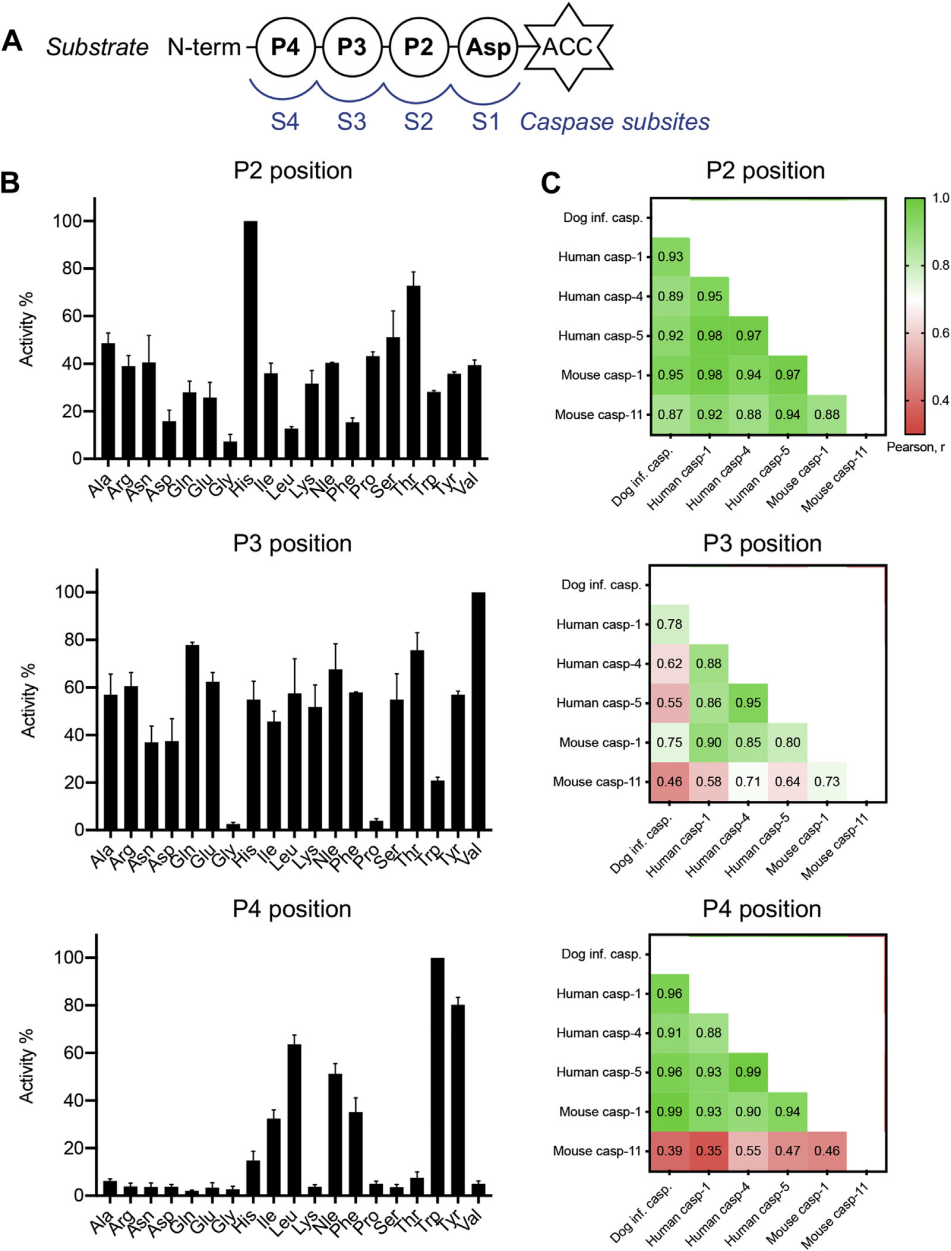
**The dog inflammatory caspase has caspase-1-like specificity.***A*, representation of interactions between the caspase substrate recognition subsites and the substrate positions P2–P4. Substrates contain Asp at P1 and are flanked at the C terminus by the ACC fluorophore. *B*, recombinant dog inflammatory caspase was screened against peptide libraries to assess the individual preferences for the P2–P4 sites. The results are presented as activity relative to the preferred amino acid at each position, as average and SD of two separate scans. *C*, Pearson correlation analysis of the relative activities of dog, human, and mouse inflammatory caspases against the P2–P4 peptide libraries. Data for mouse inflammatory caspases were obtained from Ref. (22). ACC, 7-amino-4-carbamoylmethylcoumarin.

We employed positional scanning tetrapeptide libraries to further explore this concept and reveal subtle differences between closely related enzymes (32). The dog inflammatory caspase was tolerant of a broad range of amino acids, and it preferred His in position P2 and Val in position P3. It also demonstrated the typical preference of inflammatory caspases for bulky and hydrophobic amino acids in the P4 position favoring Trp and Tyr (Fig. 3*B*).

Exploring of the specificity determinants in more depth revealed that the dog inflammatory caspase showed the highest correlation with human and mouse caspase-1 within the recognition motifs (Fig. 3*C*). Consequently, our prediction that the catalytic domain of Carnivora inflammatory caspase would have a caspase-4-like specificity is incorrect, and it has a caspase-1-like specificity.

### Resurrection of a Carnivora inflammatory caspase ancestor

**Figure 4:**
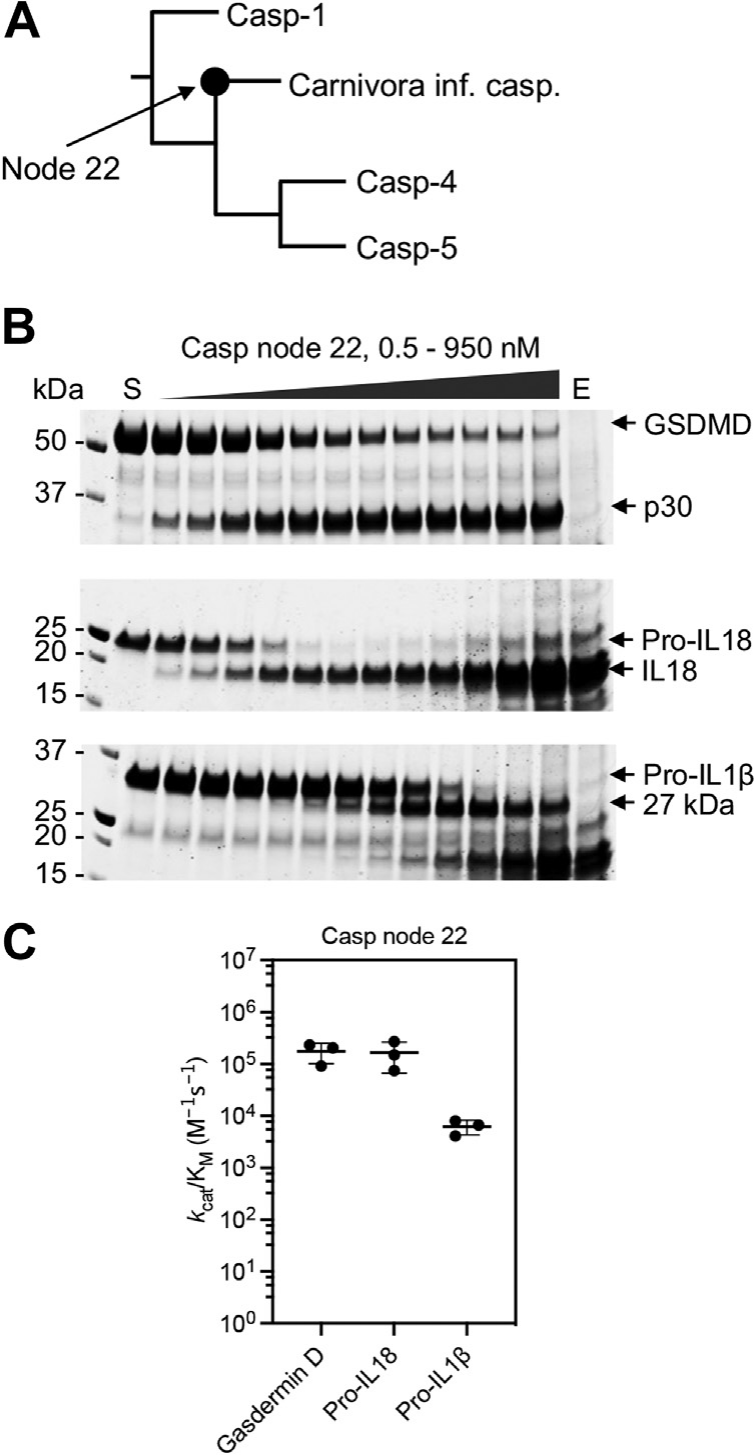
**Resurrection of an ancestral Carnivora caspase.***A*, snapshot covering the ancestral node from the phylogenetic tree (Fig. S4) used to resurrect the antecedent of the inflammatory caspase catalytic domain of the order Carnivora (caspase node 22). *B*, cleavage of protein substrates by caspase node 22. Recombinant protein substrates—mouse 4 μM gasdermin D, pro-IL-18, and pro-IL-1β—were cleaved *in vitro* with a dilution series of recombinant caspase node 22 and analyzed by Coomassie blue–stained SDS-PAGE. S and E represent the substrate- and enzyme-only controls. *C*, the catalytic parameter, *k*_cat_/K_m_, of caspase node 22 against protein substrates was calculated based on this SDS-PAGE analysis. The figure shows average and SD of three experiments. Pro-IL-1β, inactive precursor of interleukin-1β; pro-IL-18, inactive precursor of interleukin-18.

Interested in the evolutionary process that generated the caspase-1 function on the caspase-4 catalytic domain scaffold, we utilized ancestral sequence reconstruction (ASR) to examine the characteristics of the protein from which the Carnivora inflammatory caspases descended. ASR calculates a phylogenetic tree based on statistical analysis of sequence conservation and substitutions of existing proteins within a family (33). These relationships allow for calculation of sequences that represent the diverging nodes within the phylogenetic tree and thus the ancestor of each branch. The resurrected protein represents the node from which the Carnivora inflammatory caspases diverged. We termed this caspase “node 22” based on the position on the phylogenetic tree (Figs. 4*A* and S4).

**Figure 5:**
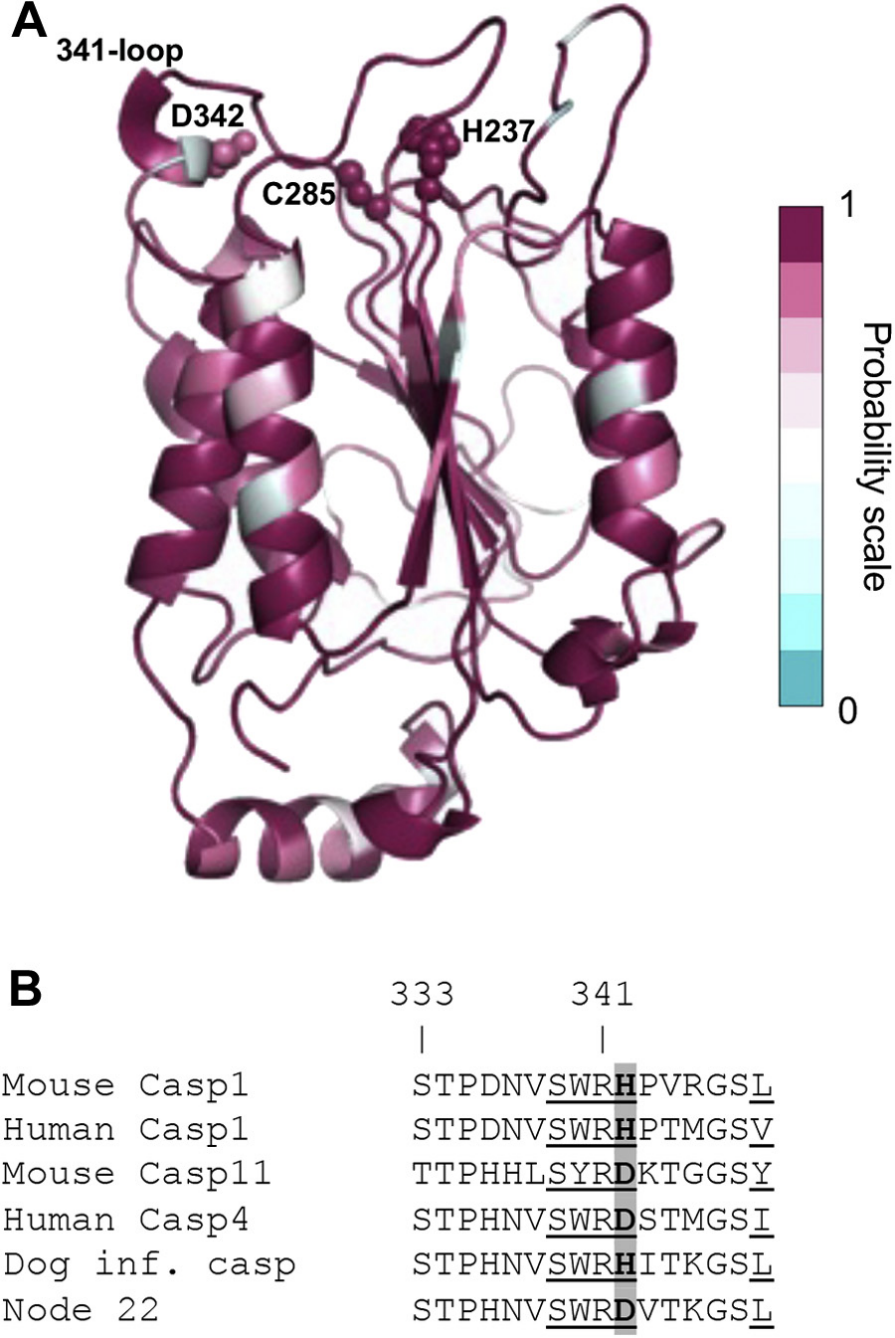
**Site-specific probabilities of caspase node 22 and comparison with extant inflammatory caspases.***A*, caspase node 22 structural model was obtained by using the Phyre2 portal (56), and each protein site was colored according to the site-specific posterior probability. The catalytic residues His-237 and Cys-285 as well as Asp-342 are shown as *spheres*. *B*, sequence alignment of the 341-loop of extant inflammatory caspases and node 22. Substrate-interacting residues of the 341-loop are underlined (43). Residue 342 is a major difference within inflammatory caspases and is highlighted in *gray*. The caspase-1 numbering convention is used.

The results of ASR analysis are site-specific probabilities for each position in the protein sequence (33, 34). Only 5% of the node 22 caspase sequence was identified as ambiguous, defined as sites with <70% probability (Fig. 5*A*). All ambiguous residues in the node 22 sequence are indicated in Fig. S5. The node 22 sequence shares more than 80% identity with the dog inflammatory caspase and human caspase-4 but only 60% identity with human caspase-1 (Fig. S5). Because node 22 is the predicted ancestor of the catalytic domain of the Carnivora inflammatory caspases, we expected that these proteins would have the same specificity. Accordingly, we hypothesized that the Carnivora inflammatory caspase evolved from a protein that should have been able to convert pro-IL-1β.

To test the specificity of node 22, we examined the cleavage efficiency on protein and peptide substrates. Node 22 appeared to have similar cleavage efficiencies of inflammatory substrates to the dog inflammatory caspase (Figs. 2 and 4, *B* and *C*). However, close observation highlights a significant distinction in the cleavage pattern of pro-IL-1β (Fig. 4*B*). While pro-IL-1β is preferably converted into the 17 kDa active form by caspase-1 (9, 22), the major observed proteolytic product of caspase node 22 was 27 kDa, an alternative product of unknown function and that can also be generated by caspase-1 (35).

**Figure 6:**
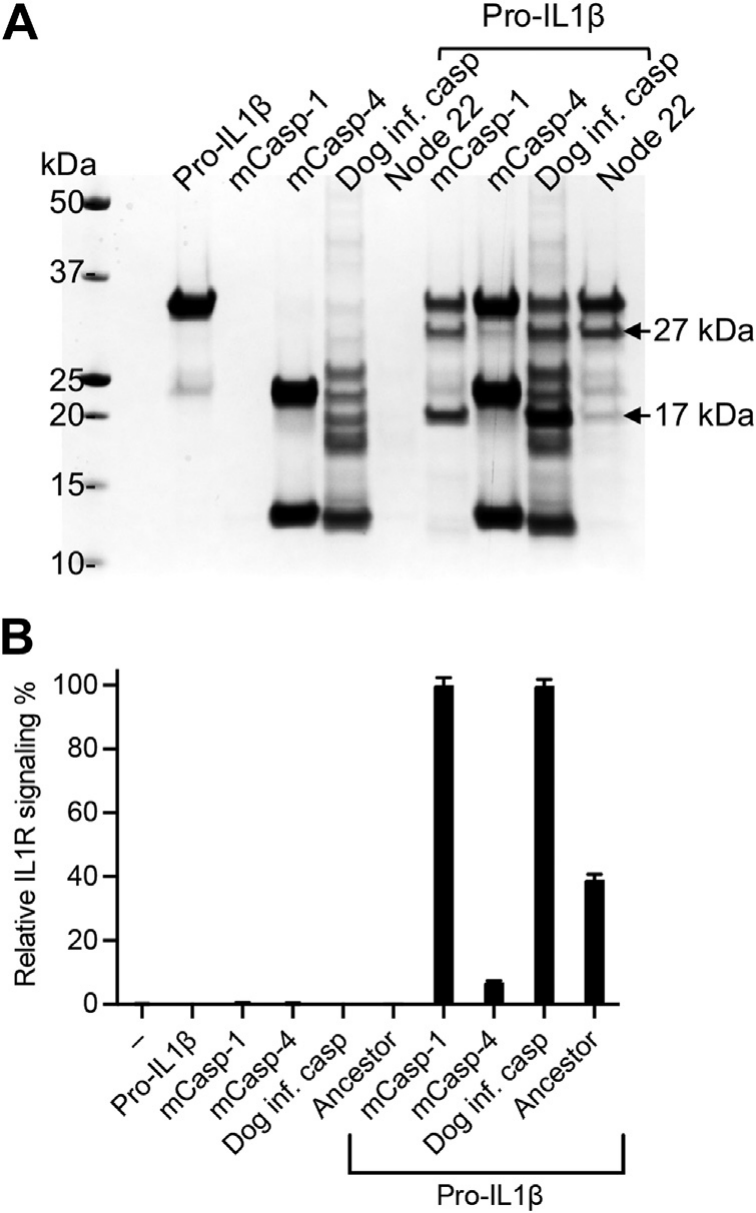
**Caspase node 22 has decreased IL-1-convertase activity.***A*, cleavage of pro-IL-1β by 40 nM mouse caspase-1, dog inflammatory caspase, and caspase node 22, or 4.5 μM mouse caspase-11. *B*, the cleavage products of pro-IL-1β from (*A*) were analyzed for biological activity using an IL-1 receptor cellular reporter assay. Data are presented as mean and SD of triplicate assays aligned under the respective cleavage reactions shown in (*A*). IL-1, interleukin 1; pro-IL-1β, inactive precursor of interleukin-1β.

To investigate whether caspase node 22 can produce a bioactive IL-1β, we used a cellular reporter assay. When we cleaved pro-IL-1β with equal amounts of caspases (Fig. 6*A*), we observed similar levels of IL-1β signaling generated by mouse caspase-1 and the dog inflammatory caspase (Fig. 6*B*). Conversely, we observed 2.5-fold lower IL-1β signaling by caspase node 22 compared with caspase-1 (Fig. 6*B*). Mouse caspase-11 served as a negative control. These results signify that caspase node 22 has decreased ability to produce bioactive IL-1β compared with caspase-1 and the dog inflammatory caspase, implying that it has caspase-4-like specificity. If this is the case, the specificity of caspase node 22 on synthetic peptides should match caspase-4. Indeed, the catalytic efficiency of caspase node 22 toward the inflammatory caspase reference substrate WEHD-7-amino-4-trifluoromethylcoumarin (AFC) was highly efficient. However, cleavage of caspase-1-optimized substrates by node 22 was substantially less efficient than cleavage by the dog inflammatory caspase (Table 2 and Fig. S6). We conclude that a caspase-4-like protease that lacked the ability to convert pro-IL-1β gave rise to the inflammatory caspase of extant Carnivora.

**Table 2:** *k*_cat_/*K*_*m*_ of the dog inflammatory caspase and resurrected ancestral caspase of node 22 for Ac-WEHD-AFC and caspase-1-optimized substrates (Ac-WQPD-ACC and Ac-FEAD-ACC)

Substrate	Dog inflammatory caspase			Ancestral caspase node 22		
	*k*_cat_ (s^−1^)	*K*_*m*_ (μM)	10^3^ × *k*_cat_/*K*_*m*_ (s^−1^ M^−1^)	*k*_cat_ (s^−1^)	*K*_*m*_ (μM)	10^3^ × *k*_cat_/*K*_*m*_ (s^−1^ M^−1^)
Ac-WEHD-AFC	8.87 ± 0.18	38 ± 1.7	233 ± 5.4	9.2 ± 0.17	17 ± 0.12	533 ± 5.8
Ac-WQPD-ACC	2.96 ± 0.26	16 ± 1.5	180 ± 0.26	1.53 ± 0.55	73 ± 37	21.7 ± 3.5
Ac-FEAD-ACC	1.22 ± 0.02	21 ± 0.81	57 ± 3.4	0.58 ± 0.03	19.2 ± 0.03	30 ± 1.8

Data show average and SD of two independent experiments.

## Discussion

The innate immune response is important in the control of infection; however, the very mechanisms that lead to its activation can also result in damaging responses to the host, such as cytokine storm, also known as hypercytokinemia (36). Host responses such as this can be lethal and thereby lead to loss of inflammatory genes through selective pressure. For example, some mammals have suffered from dampened immune responses against pathogens by downmodulating their IL-1β-converting capacity (29, 37, 38) or other innate immune–sensing mechanisms (29, 39, 40, 41). Given the importance of inflammatory caspases in programing innate immunity, we sought to understand the evolution of this response. The loss of caspase-1 in the Carnivora ancestor may represent an adaptation of this kind.

The catalytic domain of caspase-1 is absent in Carnivora and thus was lost in an early ancestor of this clade *via* the deletion of a chromosomal segment encompassing this domain, leaving caspase-4 as the single catalytically competent inflammatory caspase at this locus (28). The event that caused the loss of caspase-1 likely occurred in an early Carnivora ancestor 62 to 34 million years ago, while the clade was radiating from its origins (42). Seeking to construct the most likely sequence of the last Carnivora inflammatory caspase ancestor (node 22), we utilized every existing Carnivora inflammatory caspase catalytic domain sequence and performed ASR.

Our data suggest that caspase node 22 had the catalytic profile of current day caspase-4. This resurrected caspase would have been able to induce pyroptosis through cleavage of gasdermin D but could not produce biologically active IL-1β. This finding indicates that the early Carnivora could likely mount the first step of innate response *via* pyroptosis and release DAMPs but could not amplify the response by producing active IL-1β. However, during the evolution of the Carnivora, this caspase became more caspase-1 like, such that the current day Carnivora possess a caspase-1-like activity on a caspase-4 scaffold. Consequently, Carnivora may activate pyroptosis and amplify the response with cytokine production by using a repurposed caspase-4.

Seeking to understand the features that may drive the specificity of caspase-1 on a caspase-4 scaffold, we compared protein sequences between inflammatory caspases in the 341-loop that contacts the substrate upon proteolysis (43) (Fig. 5*B*). Of notice is His342, which is conserved in caspase-1, whereas caspase-4 shows Asp at this position. Interestingly, Asp-342 changed to an opposite charge to a histidine residue, going from caspase node 22 to the dog inflammatory caspase. The IL-1β-converting function of caspase-1 requires His342 (30). Hence, our study supports the model where His342 forms part of the pro-IL-1β recognition mechanism in caspase-1 (30).

**Figure 7:**
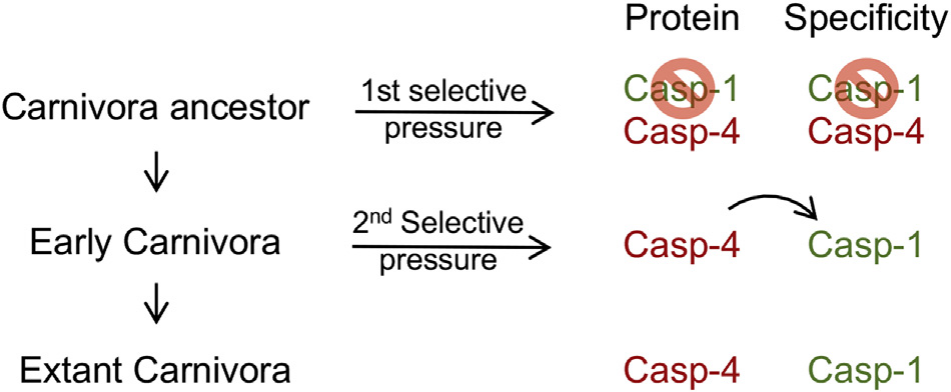
**A hypothetical process in the evolution of the Carnivora inflammatory caspase.** Before the Carnivora, there were two inflammatory caspases, caspase-1 and caspase-4. Caspase-1 was lost in a Carnivora ancestor possibly upon a first selective pressure for which production of biologically active IL-1β was detrimental. Later, an early Carnivora encountered other selective pressures that required the IL-1β response, and caspase-4 gained this activity. Extant Carnivora possess an inflammatory caspase with caspase-4 sequence homology but caspase-1 catalytic function. IL-1β, interleukin-1β.

We reason that the absence of caspase-1 in the Carnivora ancestor suppressed detrimental immune responses perhaps associated with noxious infections (Fig. 7). Survival may have depended on the loss of pro-IL-1β processing by caspase-1, while the action of caspase-4 retained the essential DAMP-releasing function of pyroptosis. We postulate that this conversion of inflammatory initiating events exemplifies the “Black Queen Hypothesis,” which proposes how natural selection favors the loss of genes that can be deleterious to the host, if other genes provide a backup function for the gene lost (44, 45).

Loss of caspase-1 in the Carnivora ancestor also represents the loss of IL-1β-conversion capacity. However, this function was regained upon modification of the caspase-4 scaffold in Carnivora, paralleling the evolution of *bona fide* caspase-1 in other species. Perhaps, resistance to other pathogenic insults encountered by the early Carnivora required the IL-1β-conversion function. Regaining this function was probably achieved by modifying the functionally closest gene, *CASP4* (Fig. 7). Thus, the caspase-4 catalytic domain with caspase-1 catalytic function became the primary inflammatory caspase in Carnivora. Several genes involved in inflammatory cell death have been lost or are present as pseudogenes in Carnivorans (29, 46). Hence, modification of the inflammatory caspase locus represents one example of how mammals of this order have coped with the unfavorable side effects of inflammation throughout their evolution.

## Experimental procedures

### Plasmids

Constructs encoding mouse pro-IL-18 and pro-IL-1β in pET29b(+) and mouse gasdermin D in pET15b were previously described (9, 22). Deletion of the N-terminal CARD of inflammatory caspases results in a form that encompasses only the catalytic domain. The constructs encoding the CARD-depleted versions of the human and mouse inflammatory caspases were previously described (22). The nucleotide sequence encoding the ORF of the dog inflammatory caspase, corresponding to UniProt Q9MZV7 limits 132 to 404, was codon optimized for *Escherichia coli* expression and cloned NdeI/XhoI into pET29b(+). The nucleotide sequence of the resurrected ancestral protein of the Carnivora clade, caspase node 22, was cloned with the same strategy.

### Protein expression and purification

BL21(DE3)-competent *E. coli* were utilized to transform all constructs. Bacterial cells were cultured in 2xYT media containing appropriate antibiotics and expressed and purified as described previously (47). Caspase of node 22 was expressed and purified following the same method. Protein concentrations were calculated by absorbance at 280 nm. The concentration of active proteases was determined by active-site titration assays against the pan-caspase inhibitor, benzyloxycarbonyl—Val-Ala-Asp–fluoromethyl ketone. The remaining activity was measured with Ac-WEHD-AFC (100 μM) substrate in caspase assay buffer 20 mM Pipes (pH 7.2), 10% sucrose, 100 mM NaCl, 1 mM EDTA, and 10 mM DTT supplemented with 0.75 M sodium citrate (48).

### Fluorescent substrates

Internally quenched fluorescent substrates flanked by the N-terminal fluorophore 7-amino-4-carbamoylmethylcoumarin (ACC) and a C-terminal quencher 2,4-dinitrophenyl-lysine were synthesized by solid-phase peptide synthesis as previously described (9, 49). The ACC fluorophore–containing substrates Ac-WQPD-ACC and Ac-FEAD-ACC were synthesized as described (22). All in-house synthesized substrates were purified using reversed-phase HPLC, dissolved in dimethyl sulfoxide to the concentration of 10 mM, and stored at −20 °C until use. The AFC fluorophore–containing substrate Ac-WEHD-AFC was obtained from Enzo Life Sciences.

### Activity assays

Enzymatic assays of recombinant caspases consisted of 100 μl final volume and were performed in 96-well opaque plates (Costar, Corning). Caspases were incubated in caspase assay buffer for 10 min at 37 °C before measuring activity, which was initiated upon addition of substrate. Activity assays consisted of kinetic fluorescence measurement in a CLARIOstar plate reader (BMG LabTech). The ACC fluorophore was detected at excitation/emission 355/460 nm and AFC at 400/505 nm. Reaction velocity was defined as the linear portion of the kinetic curve (relative fluorescence unit/second). Relative fluorescence unit values were converted to concentration by comparison with known standards.

Specificity parameters of caspases for the fluorogenic substrates were determined from enzyme kinetic data of 30 min reactions. The reaction velocity was plotted against substrate concentration, and *V*_max_ and K_m_ were calculated with Prism 7 (GraphPad Software, Inc) using the Michaelis–Menten equation. *k*_cat_ was obtained by using Equation 1, where [E] is the enzyme concentration measured by active-site titration.

(1) $$k_{\text{cat}}=V_{\max}/\left[\text{E}\right]$$

### Screening of caspase specificity

A fluorogenic substrate library was utilized to define the amino acid cleavage preference of the dog inflammatory caspase, as described before for other caspases (49). Briefly, three sublibraries that scan positions surrounding the cleavage site (P2, P3, and P4) were screened at a concentration of 100 μM with the dog inflammatory caspase, human caspase-1, caspase-4, and caspase-5, in a final volume of 100 μl in caspase assay buffer. Substrate hydrolysis was monitored to determine reaction velocity. For each sublibrary, percent activity was calculated in relation to the optimal amino acid. We used the relative activity values obtained here for the dog inflammatory caspases and human inflammatory caspases, together with previously reported data for mouse inflammatory caspases (22), to compare their substrate preference by means of the Pearson correlation coefficient (*r*) using Prism 7.

### Cleavage assays of recombinant protein substrates

We used SDS-PAGE to analyze cleavage of protein substrates by the caspases. Recombinant caspases were serially diluted and incubated with 4 μM of recombinant mouse protein substrates obtained as previously described (9). Controls consisted of substrate or enzyme alone. After incubation, reactions were stopped by the addition of 30 μl of 3× SDS loading buffer and incubated at 95 °C for 5 min. Products were separated in Bolt 4 to 12% Bis–Tris SDS-PAGE (Thermo Fisher Scientific) and stained with InstantBlue (Expedeon). The gels were scanned with an ODYSSEY CLx imager (LI-COR). Images were exported to Image Studio software (LI-COR) for band intensity quantification of protein substrate remaining after cleavage. Band intensity values were normalized relative to those of noncleaved substrate, and values were plotted against log [E] estimated by active-site titration. E_1/2_ values were calculated with Prism 7 using the log(inhibitor) *versus* response (three parameters) equation. E_1/2_ values were used to calculate the catalytic efficiency of caspases for protein substrates according to Equation 2 (48). Where *k*_cat_/*K*_*m*_ is the second-order rate constant for substrate hydrolysis, E_1/2_ is the concentration of caspase for 50% hydrolysis of substrate, and *t* is the reaction time in seconds.

(2) $$k_{cat}/K_{M}=\ln\;2/\left({\text{E}}_{1/2}\times t\right)$$

### Phylogenetic trees and computation of ancestral sequences

Seeking proteins with the caspase CARD–CARD–catalytic domain arrangement, we used BLAST on UniProt (50), Ensembl (51), and the National Center for Biotechnology Information to retrieve related proteins employing the dog inflammatory caspase (UniProt: A9YEF4) as a query. The origin of caspase-4 is found in early mammals; hence, we focused on Mammalia in our homology search. To resurrect a highly probable sequence of the last common ancestor of the Carnivora clade (node 22), we utilized a database of curated caspase sequences (CaspBase.org) that provided inflammatory caspase protein sequences from the chordate lineage (52) (Table S2). PROMALS3D (prodata.swmed.edu/promals3d) generated structure-based alignments (53), and sequences were pruned on Jalview (jalview.org) (54) to remove the CARDs so that we could focus our analysis on the catalytic domain. Finally, ancestral protein reconstruction proceeded as previously described by Grinshpon *et al*. (55). Structural model of the ancestral reconstructed caspase was obtained by the PHYRE2 protein fold recognition server (http://www.sbg.bio.ic.ac.uk/∼phyre2/html/page.cgi?id=index) (56).

### IL-1 receptor signaling assay

To measure signaling through the IL-1 receptor by IL-1β proteolytic products, we utilized IL-1β reporter human embryonic kidney 293 cells with an NF-κF06BB-inducible secreted embryonic alkaline phosphatase (InVivoGen; catalog no.: hkb-il1bv2). The reporter cells were cultured in Dulbecco's modified Eagle's medium, 4.5 g/l glucose, 2 mM l-glutamine, 10% (v/v) heat-inactivated fetal bovine serum, 100 U/ml penicillin, 100 μg/ml streptomycin, 100 μg/ml normocin, and 100 μg/ml zeocin (selective antibiotic). For the assay, pro-IL-1β (4.25 μM) was incubated with 40 nM mouse caspase-1, dog inflammatory caspase, and caspase node 22, or 4.25 μM mouse caspase-11, for 30 min in caspase assay buffer. Upon incubation, reaction products were diluted to 20 nM IL-1β in test media (culture media without normocin or zeocin). A total of 50 μl of diluted IL-1β products were transferred to a 96-well tissue culture–treated plate and mixed with 5 × 10^4^ IL-1β reporter cells in test media and incubated for 20 h. After incubation, secreted embryonic alkaline phosphatase was measured by utilizing 20 μl of IL-1β reporter cell supernatants and mixed with 180 μl of Quanti-Blue solution (InVivoGen; catalog no.: rep-qbs) and incubated at 37 °C for 30 min. Absorbance was measured at 630 nm in a CLARIOstar plate reader and normalized to signal obtained with caspase-1-cleaved IL-1β.

## Data availability

All data generated and analyzed during this study are included in this published article and its supporting information file.

## Supporting information

This article contains supporting information.

## Conflict of interest

The authors declare that they have no conflicts of interest with the contents of this article.

## References

[1] Salvesen G.S., Hempel A., Coll N.S. (2016). Protease signaling in animal and plant-regulated cell death. FEBS J..

[2] Martin S.J., Henry C.M., Cullen S.P. (2012). A perspective on mammalian caspases as positive and negative regulators of inflammation. Mol. Cell.

[3] Van Opdenbosch N., Lamkanfi M. (2019). Caspases in cell death, inflammation, and disease. Immunity.

[4] Cookson B.T., Brennan M.A. (2001). Pro-inflammatory programmed cell death. Trends Microbiol..

[5] Man S.M., Karki R., Kanneganti T.D. (2017). Molecular mechanisms and functions of pyroptosis, inflammatory caspases and inflammasomes in infectious diseases. Immunol. Rev..

[6] Martinon F., Burns K., Tschopp J. (2002). The inflammasome: A molecular platform triggering activation of inflammatory caspases and processing of proIL-b. Mol. Cell.

[7] Kayagaki N., Warming S., Lamkanfi M., Vande Walle L., Louie S., Dong J., Newton K., Qu Y., Liu J., Heldens S., Zhang J., Lee W.P., Roose-Girma M., Dixit V.M. (2011). Non-canonical inflammasome activation targets caspase-11. Nature.

[8] Vigano E., Diamond C.E., Spreafico R., Balachander A., Sobota R.M., Mortellaro A. (2015). Human caspase-4 and caspase-5 regulate the one-step non-canonical inflammasome activation in monocytes. Nat. Commun..

[9] Bibo-Verdugo B., Snipas S.J., Kolt S., Poreba M., Salvesen G.S. (2020). Extended subsite profiling of the pyroptosis effector protein gasdermin D reveals a region recognized by inflammatory caspase-11. J. Biol. Chem..

[10] Kayagaki N., Stowe I.B., Lee B.L., O'Rourke K., Anderson K., Warming S., Cuellar T., Haley B., Roose-Girma M., Phung Q.T., Liu P.S., Lill J.R., Li H., Wu J., Kummerfeld S. (2015). Caspase-11 cleaves gasdermin D for non-canonical inflammasome signalling. Nature.

[11] Shi J., Zhao Y., Wang K., Shi X., Wang Y., Huang H., Zhuang Y., Cai T., Wang F., Shao F. (2015). Cleavage of GSDMD by inflammatory caspases determines pyroptotic cell death. Nature.

[12] Ding J., Wang K., Liu W., She Y., Sun Q., Shi J., Sun H., Wang D.C., Shao F. (2016). Pore-forming activity and structural autoinhibition of the gasdermin family. Nature.

[13] Liu X., Zhang Z., Ruan J., Pan Y., Magupalli V.G., Wu H., Lieberman J. (2016). Inflammasome-activated gasdermin D causes pyroptosis by forming membrane pores. Nature.

[14] Evavold C.L., Ruan J., Tan Y., Xia S., Wu H., Kagan J.C. (2018). The pore-forming protein gasdermin D regulates interleukin-1 secretion from living macrophages. Immunity.

[15] Heilig R., Dick M.S., Sborgi L., Meunier E., Hiller S., Broz P. (2018). The Gasdermin-D pore acts as a conduit for IL-1β secretion in mice. Eur. J. Immunol..

[16] Monteleone M., Stanley A.C., Chen K.W., Brown D.L., Bezbradica J.S., von Pein J.B., Holley C.L., Boucher D., Shakespear M.R., Kapetanovic R., Rolfes V., Sweet M.J., Stow J.L., Schroder K. (2018). Interleukin-1β maturation triggers its relocation to the plasma membrane for gasdermin-D-dependent and -independent secretion. Cell Rep..

[17] Xia S., Zhang Z., Magupalli V.G., Pablo J.L., Dong Y., Vora S.M., Wang L., Fu T.-M., Jacobson M.P., Greka A., Lieberman J., Ruan J., Wu H. (2021). Gasdermin D pore structure reveals preferential release of mature interleukin-1. Nature.

[18] Akita K., Ohtsuki T., Nukada Y., Tanimoto T., Namba M., Okura T., Takakura-Yamamoto R., Torigoe K., Gu Y., Su M.S.-S., Fujii M., Satoh-Itoh M., Yamamoto K., Kohno K., Ikeda M. (1997). Involvement of caspase-1 and caspase-3 in the production and processing of mature human interleukin 18 in monocytic THP.1 cells. J. Biol. Chem..

[19] Gu Y., Kuida K., Tsutsui H., Ku G., Hsiao K., Fleming M.A., Hayashi N., Higashino K., Okamura H., Nakanishi K., Kurimoto M., Tanimoto T., Flavell R.A., Sato V., Harding M.W. (1997). Activation of interferon-γ inducing factor mediated by interleukin-1β converting enzyme. Science.

[20] Mosley B., Urdal D.L., Prickett K.S., Larsen A., Cosman D., Conlon P.J., Gillis S., Dower S.K. (1987). The interleukin-1 receptor binds the human interleukin-1 alpha precursor but not the interleukin-1 beta precursor. J. Biol. Chem..

[21] Sims J.E., Smith D.E. (2010). The IL-1 family: Regulators of immunity. Nat. Rev. Immunol..

[22] Ramirez M.L.G., Poreba M., Snipas S.J., Groborz K., Drag M., Salvesen G.S. (2018). Extensive peptide and natural protein substrate screens reveal that mouse caspase-11 has much narrower substrate specificity than caspase-1. J. Biol. Chem..

[23] Rühl S., Broz P. (2015). Caspase-11 activates a canonical NLRP3 inflammasome by promoting K+ efflux. Eur. J. Immunol..

[24] Dinarello C.A. (2018). Overview of the IL-1 family in innate inflammation and acquired immunity. Immunol. Rev..

[25] Garlanda C., Dinarello Charles A., Mantovani A. (2013). The interleukin-1 family: Back to the future. Immunity.

[26] Ramirez M.L.G., Salvesen G.S. (2018). A primer on caspase mechanisms. Semin. Cell Dev. Biol..

[27] Martinon F., Tschopp J. (2007). Inflammatory caspases and inflammasomes: Master switches of inflammation. Cell Death Differ..

[28] Eckhart L., Ballaun C., Hermann M., VandeBerg J.L., Sipos W., Uthman A., Fischer H., Tschachler E. (2008). Identification of novel mammalian caspases reveals an important role of gene loss in shaping the human caspase repertoire. Mol. Biol. Evol..

[29] Digby Z., Tourlomousis P., Rooney J., Boyle J.P., Bibo-Verdugo B., Pickering R.J., Webster S.J., Monie T.P., Hopkins L.J., Kayagaki N., Salvesen G.S., Warming S., Weinert L., Bryant C.E. (2021). Evolutionary loss of inflammasomes in the Carnivora and implications for the carriage of zoonotic infections. Cell Rep..

[30] Devant P., Cao A., Kagan J.C. (2021). Evolution-inspired redesign of the LPS receptor caspase-4 into an interleukin-1β–converting enzyme. Sci. Immunol..

[31] Bibo-Verdugo B., Salvesen G.S. (2022). Caspase mechanisms in the regulation of inflammation. Mol. Aspects Med..

[32] Thornberry N.A., Rano T.A., Peterson E.P., Rasper D.M., Timkey T., Margarita G.-C., Houtzager V.M., Nordstrom P.A., Roy S., Viaillancourt J.P., Chapman K.T., Nicholson D.W. (1997). A combinatorial approach defines specificities of members of the caspase family and granzyme B. J. Biol. Chem..

[33] Harms M.J., Thornton J.W. (2010). Analyzing protein structure and function using ancestral gene reconstruction. Curr. Opin. Struct. Biol..

[34] Eick G.N., Bridgham J.T., Anderson D.P., Harms M.J., Thornton J.W. (2016). Robustness of reconstructed ancestral protein functions to statistical uncertainty. Mol. Biol. Evol..

[35] Afonina I.S., Muller C., Martin S.J., Beyaert R. (2015). Proteolytic processing of interleukin-1 family cytokines: Variations on a common theme. Immunity.

[36] Wong J.P., Viswanathan S., Wang M., Sun L.-Q., Clark G.C., D'Elia R.V. (2017). Current and future developments in the treatment of virus-induced hypercytokinemia. Future Med. Chem..

[37] Cui H., Zhang L. (2021). Key components of inflammasome and pyroptosis pathways are deficient in canines and felines, possibly affecting their response to SARS-CoV-2 infection. Front. Immunol..

[38] Goh G., Ahn M., Zhu F., Lee L.B., Luo D., Irving A.T., Wang L.-F. (2020). Complementary regulation of caspase-1 and IL-1β reveals additional mechanisms of dampened inflammation in bats. Proc. Natl. Acad. Sci. U. S. A..

[39] Xie J., Li Y., Shen X., Goh G., Zhu Y., Cui J., Wang L.-F., Shi Z.-L., Zhou P. (2018). Dampened STING-dependent interferon activation in bats. Cell Host & Microbe.

[40] Fischer H., Tschachler E., Eckhart L. (2020). Cytosolic DNA sensing through cGAS and STING is inactivated by gene mutations in pangolins. Apoptosis.

[41] Fischer H., Tschachler E., Eckhart L. (2020). Pangolins lack IFIH1/MDA5, a cytoplasmic RNA sensor that initiates innate immune defense upon coronavirus infection. Front. Immunol..

[42] Martin L.D., Gittleman J.L. (1989). Carnivore Behavior, Ecology, and Evolution.

[43] Fuentes-Prior P., Salvesen G.S. (2004). The protein structures that shape caspase activity, specificity, activation and inhibition. Biochem. J..

[44] Albalat R., Cañestro C. (2016). Evolution by gene loss. Nat. Rev. Genet..

[45] Morris J.J., Lenski R.E., Zinser E.R. (2012). The black queen hypothesis: Evolution of dependencies through adaptive gene loss. mBio.

[46] Dondelinger Y., Hulpiau P., Saeys Y., Bertrand M.J.M., Vandenabeele P. (2016). An evolutionary perspective on the necroptotic pathway. Trends Cell Biol..

[47] Denault J.B., Salvesen G.S. (2003). Expression, purification, and characterization of caspases. Curr. Protoc. Protein Sci..

[48] Stennicke H.R., Salvesen G.S. (2000). Caspase Assays.

[49] Poreba M., Kasperkiewicz P., Snipas S.J., Fasci D., Salvesen G.S., Drag M. (2014). Unnatural amino acids increase sensitivity and provide for the design of highly selective caspase substrates. Cell Death Differ..

[50] Consortium T.U. (2020). UniProt: The universal protein knowledgebase in 2021. Nucl. Acids Res..

[51] Howe K.L., Achuthan P., Allen J., Allen J., Alvarez-Jarreta J., Amode M.R., Armean I.M., Azov A.G., Bennett R., Bhai J., Billis K., Boddu S., Charkhchi M., Cummins C., Da Rin Fioretto L. (2020). Ensembl 2021. Nucl. Acids Res..

[52] Grinshpon R.D., Williford A., Titus-McQuillan J., Clay Clark A. (2018). The CaspBase: A curated database for evolutionary biochemical studies of caspase functional divergence and ancestral sequence inference. Protein Sci..

[53] Pei J., Grishin N.V. (2014). PROMALS3D: Multiple protein sequence alignment enhanced with evolutionary and three-dimensional structural information. Methods Mol. Biol..

[54] Waterhouse A.M., Procter J.B., Martin D.M.A., Clamp M., Barton G.J. (2009). Jalview Version 2—a multiple sequence alignment editor and analysis workbench. Bioinformatics.

[55] Grinshpon R.D., Shrestha S., Titus-McQuillan J., Hamilton P.T., Swartz P.D., Clark A.C. (2019). Resurrection of ancestral effector caspases identifies novel networks for evolution of substrate specificity. Biochem. J..

[56] Kelley L.A., Mezulis S., Yates C.M., Wass M.N., Sternberg M.J.E. (2015). The Phyre2 web portal for protein modeling, prediction and analysis. Nat. Protoc..

