# Supplementary material for: Resurrection of an ancient inflammatory locus reveals switch to caspase-1 specificity on a caspase-4 scaffold
Source: J Biol Chem. 2022 Apr 12;298(6):101931. doi: 10.1016/j.jbc.2022.101931 (PMC9144055; doi:10.1016/j.jbc.2022.101931)

Supplementary table 1. List of hybrid inflammatory caspase sequences identified by BLAST search.

	**Species name**	**Order**	**Family**	**Accession number**
1	*Canis lupus familiaris*	Carnivora	Canidae	Uniprot A9YEF4
2	*Vulpes vulpes*	Carnivora	Canidae	NCBI XP_025862547
3	*Felis catus*	Carnivora	Felidae	Uniprot A9YEF3
4	*Panthera tigris*	Carnivora	Felidae	NCBI XP_007076019
5	*Panthera pardus*	Carnivora	Felidae	NCBI XP_019290827
6	*Puma concolor*	Carnivora	Felidae	NCBI XP_025784844
7	*Acinonyx jubatus*	Carnivora	Felidae	NCBI XP_014929420
8	*Lynx pardinus*	Carnivora	Felidae	NCBI VFV34370
9	*Lynx canadensis*	Carnivora	Felidae	NCBI XP_030188912
10	*Ailuropoda melanoleuca*	Carnivora	Ursidae	Uniprot G1L0X5
11	*Ursus maritimus*	Carnivora	Ursidae	Uniprot A0A384C1R8
12	*Ursus arctos*	Carnivora	Ursidae	NCBI XP_026361660
13	*Neomonachus schauinslandi*	Carnivora	Phocidae	Uniprot A0A2Y9HK57
14	*Leptonychotes weddellii*	Carnivora	Phocidae	Uniprot A0A2U3XCR7
15	*Phoca vitulina*	Carnivora	Phocidae	NCBI XP_032284077
16	*Mirounga leonina*	Carnivora	Phocidae	NCBI XP_034864710
17	*Halichoerus grypus*	Carnivora	Phocidae	NCBI XP_035959675
18	*Enhydra lutris*	Carnivora	Mustelidae	Uniprot A0A2Y9KBG1
19	*Lontra canadensis*	Carnivora	Mustelidae	NCBI XP_032699986
20	*Zalophus californianus*	Carnivora	Otariidae	NCBI XP_027433511
21	*Eumetopias jubatus*	Carnivora	Otariidae	NCBI XP_027961789
22	*Hyaena hyaena*	Carnivora	Hyaenidae	NCBI XP_039110315
23	*Odobenus rosmarus*	Carnivora	Odobenidae	Uniprot A0A2U3ZX61
24	*Ovis aries*	Artiodactyla	Bovidae	Uniprot W5NYL5

Supplementary table 2. List of taxa and caspases used in the reconstruction of a Carnivora inflammatory caspase ancestor.

Caspase	Species
Caspase-1	*Alligator mississippiensis*
Caspase-1	*Aptenodytes forster*
Caspase-1	*Bos taurus*
Caspase-1	*Camelus bactrianus*
Caspase-1	*Camelus dromedarius*
Caspase-1	*Camelus ferus*
Caspase-1	*Callithrix jacchus*
Caspase-1	*Castor canadensis*
Caspase-1	*Cebus capucinus imitator*
Caspase-1	*Chlorocebus sabaeus*
Caspase-1	*Colobus angolensis*
Caspase-1	*Columba livia*
Caspase-1	*Cricetulus griseus*
Caspase-1	*Danio rerio*
Caspase-1	*Dasypus novemcinctus*
Caspase-1	*Delphinapterus leucas*
Caspase-1	*Echinops telfairi*
Caspase-1	*Equus asinus*
Caspase-1	*Equus caballus*
Caspase-1	*Equus przewalskii*
Caspase-1	*Fukomys damarensis*
Caspase-1	*Galeopterus variegatus*
Caspase-1	*Gallus gallus*
Caspase-1	*Gorilla gorilla gorilla*
Caspase-1	*Heterocephalus glaber*
Caspase-1	*Hipposideros armiger*
Caspase-1	*Homo sapiens*
Caspase-1	*Jaculus jaculus*
Caspase-1	*Lepisosteus oculatus*
Caspase-1	*Macaca fascicularis*
Caspase-1	*Macaca mulatta*
Caspase-1	*Macaca nemestrina*
Caspase-1	*Mandrillus leucophaeus*
Caspase-1a	*Maylandia zebra*
Caspase-1	*Melopsittacus undulatus*
Caspase-1	*Microcebus murinus*
Caspase-1	*Microtus ochrogaster*
Caspase-1	*Mus caroli*

Caspase-1	*Mus musculus*
Caspase-1	*Mus Pahari*
Caspase-1	*Myotis brandtii*
Caspase-1	*Myotis davidii*
Caspase-1	*Nannospalax galili*
Caspase-1	*Nomascus leucogenys*
Caspase-1	*Octodon degus*
Caspase-1	*Otolemur garnettii*
Caspase-1	*Orcinus orca*
Caspase-1	*Oreochromicus niloticus*
Caspase-1a	*Oreochromus niloticus*
Caspase-1	*Orycteropus afer afer*
Caspase-1	*Oryctogalus cuniculus*
Caspase-1	*Pan paniscus*
Caspase-1	*Pan troglodytes*
Caspase-1	*Papio Anubis*
Caspase-1	*Peromyscus manicula*
Caspase-1	*Pongo abellii*
Caspase-1	*Propithecus coquereli*
Caspase-1	*Pteroptus vampyrus*
Caspase-1	*Pygocentrus nattereri*
Caspase-1	*Rattus norvegicus*
Caspase-1	*Rhinopthecus bieti*
Caspase-1	*Rhinopthecus roxellana*
Caspase-1	*Rousettus aegyptiacus*
Caspase-1	*Saimiri boliviensis*
Caspase-1	*Sus scrofa*
Caspase-1	*Trichechus manatus*
Caspase-1	*Tupaia chinensis*
Caspase-1	*Tursiops truncatus*
Caspase-1	*Vicugna pacos*
Caspase-1a	*Xenopus laevis*
Caspase-1b	*Xenopus laevis*
Caspase-4	*Aotus nancymaae*
Caspase-4	*Bos taurus*
Caspase-4	*Callithrix jacchus*
Caspase-4	*Cebus capucinus imitator*
Caspase-4	*Cerocebus atys*
Caspase-4	*Chinchilla lanigera*
Caspase-4	*Chlorocebus sabaeus*
Caspase-4	*Colobus angolensis*
Caspase-4	*Equus caballus*

Caspase-4	*Gorilla gorilla gorilla*
Caspase-4	*Heterocephalus glaber*
Caspase-4	*Homo sapiens*
Caspase-4	*Jaculus jaculus*
Caspase-4	*Macaca fascicularis*
Caspase-4	*Macaca mulatta*
Caspase-4	*Macaca nemestrina*
Caspase-4	*Mandrillus leucophaeus*
Caspase-4	*Mus caroli*
Caspase-4	*Mus musculus*
Caspase-4	*Mus pahari*
Caspase-4	*Mustela putorious furo*
Caspase-4	*Myotis lucifugus*
Caspase-4	*Nannospalax galili*
Caspase-4	*Nomascus leucogenys*
Caspase-4	*Ochotona princeps*
Caspase-4	*Oryctolagus cuniculus*
Caspase-4	*Otolemur garnettii*
Caspase-4	*Ovis aries*
Caspase-4	*Pan troglodytes*
Caspase-4	*Papio anubis*
Caspase-4	*Pongo abelii*
Caspase-4	*Rattus norvegicus*
Caspase-4	*Rhinopthecus roxellana*
Caspase-4	*Saimiri boliviensis*
Caspase-4	*Sarcophilus harrisi*
Caspase-4	*Tupaia chinensis*
Caspase-5	*Dipodomys ordii*
Caspase-5	*Gorilla gorilla gorilla*
Caspase-5	*Homo sapiens*
Caspase-5	*Macaca fascicularis*
Caspase-5	*Macaca mulatta*
Caspase-5	*Macaca nemestrina*
Caspase-5	*Mandrillus leucophaeus*
Caspase-5	*Nomascus leucogenys*
Caspase-5	*Pan troglodytes*
Caspase-5	*Papio anubis*
Caspase-5	*Pongo abelii*
Caspase-5	*Rhinopthecus roxellana*
Caspase-5	*Saimiri boliviensis*

Supplementary figure 1. Cladograms of caspase activation and recruitment domains (CARDs) and catalytic domains of inflammatory caspases. The branches containing caspase-1 and caspase-4 sequences are colored in green and red, respectively. Green triangles indicate CARD1, and orange triangles represent CARD2, whereas blue dots represent the catalytic domain from hybrid inflammatory caspases.

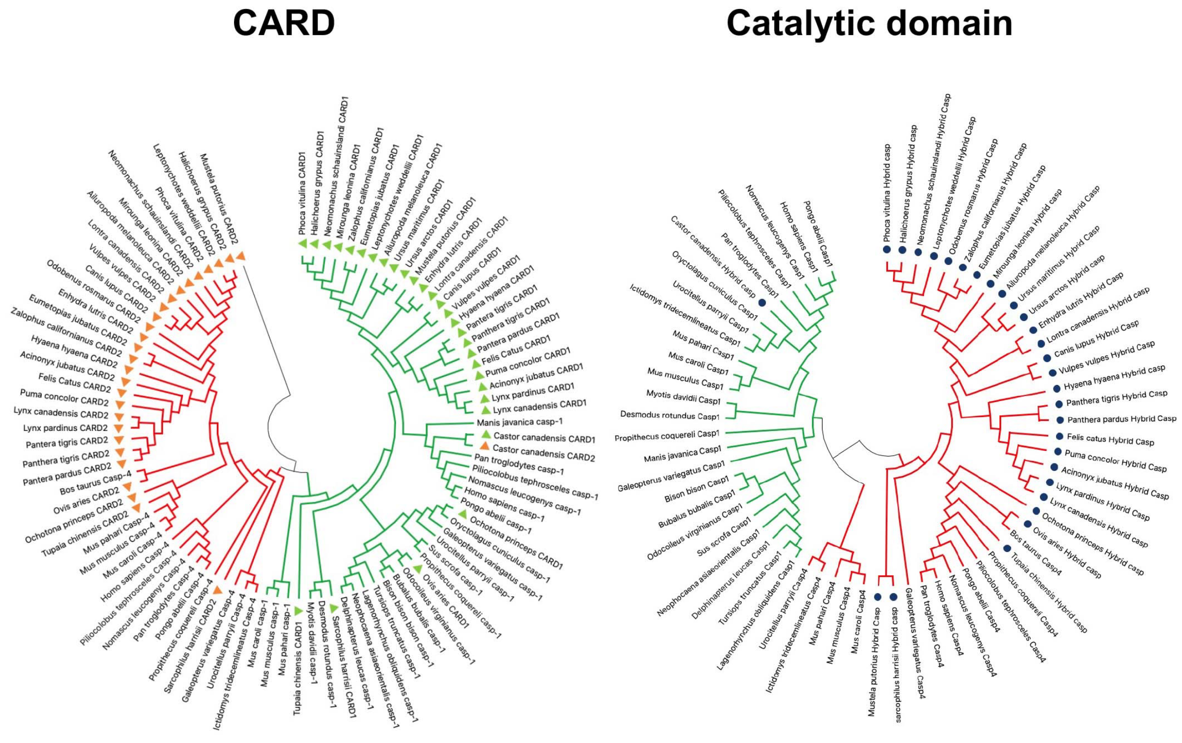

Supplementary figure 2. Purification of the dog inflammatory caspase catalytic domain. A) Recombinant dog inflammatory caspase with C-terminal 6xHis tag was expressed in bacteria and soluble intracellular proteins were Ni-affinity purified. p20 and p10 subunits are identified based on molecular weight. Fractions enclosed by the dotted line were pooled and assayed with Ac-WEHD-AFC fluorogenic substrate (B).

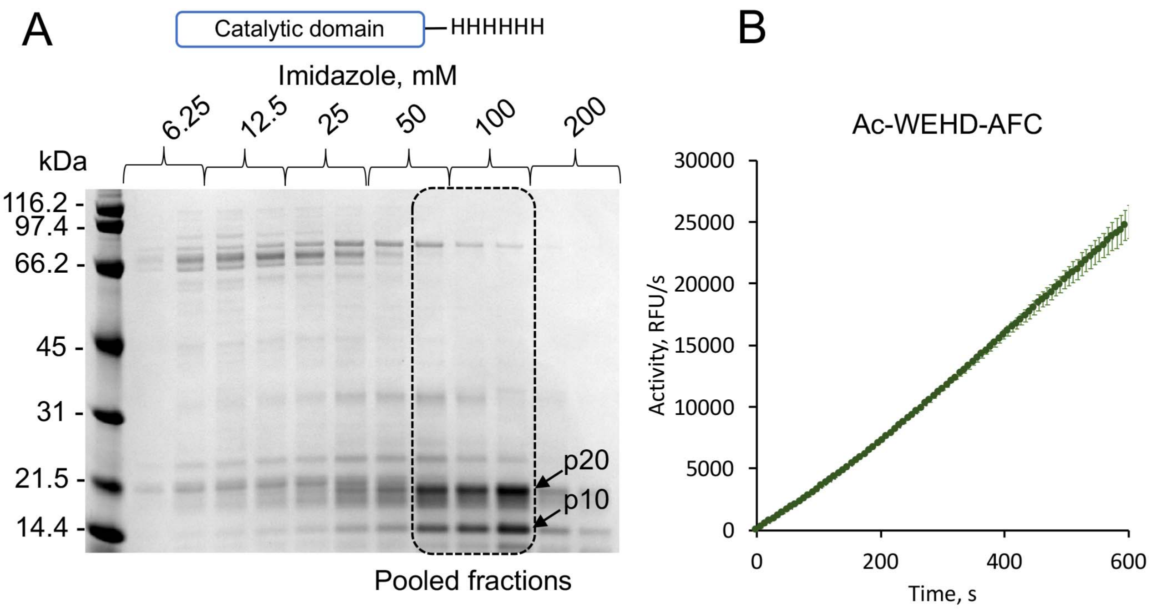

Supplementary figure 3. Substrate velocity plots were utilized to calculate k_{cat}/K_M (related to table 1).

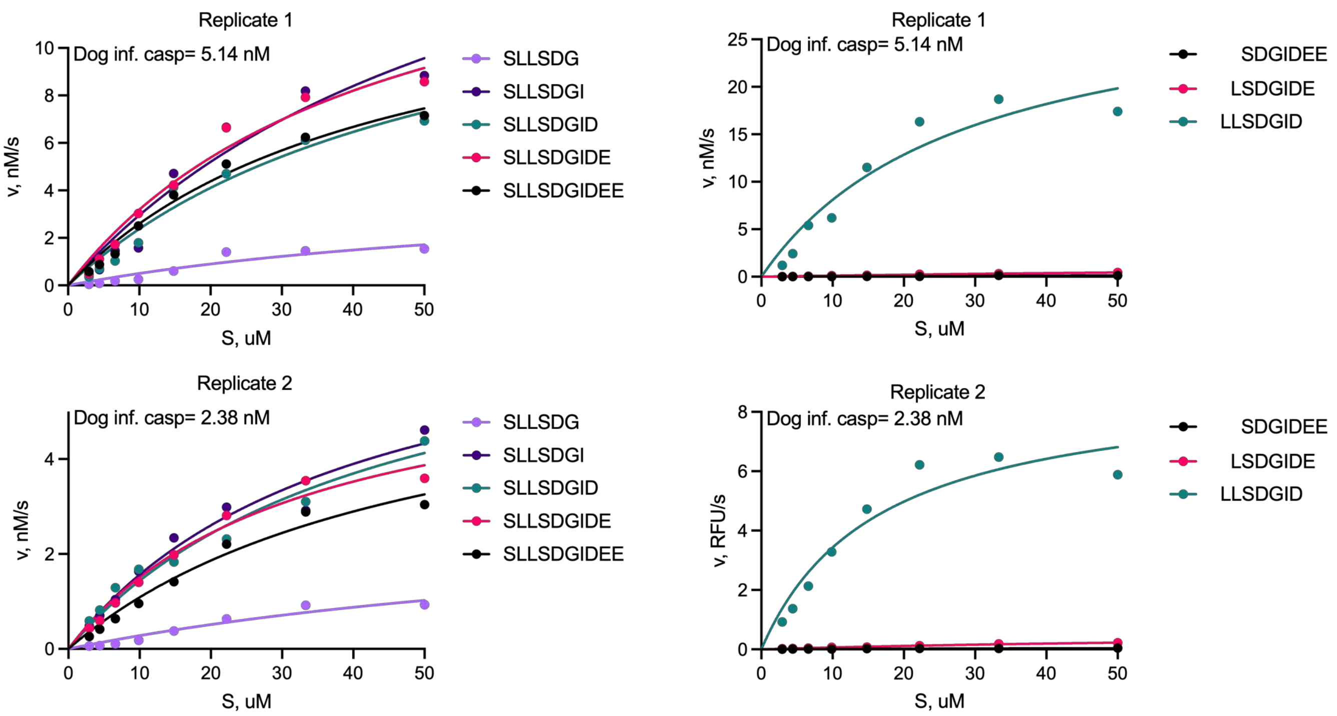

Supplementary figure 4. Phylogenetic tree of the inflammatory caspases used for the ancestral protein reconstruction. The last Carnivora inflammatory caspase ancestor, node 22, is highlighted on the red branch comprising the Carnivora inflammatory caspases, which are often annotated as hybrid caspases.

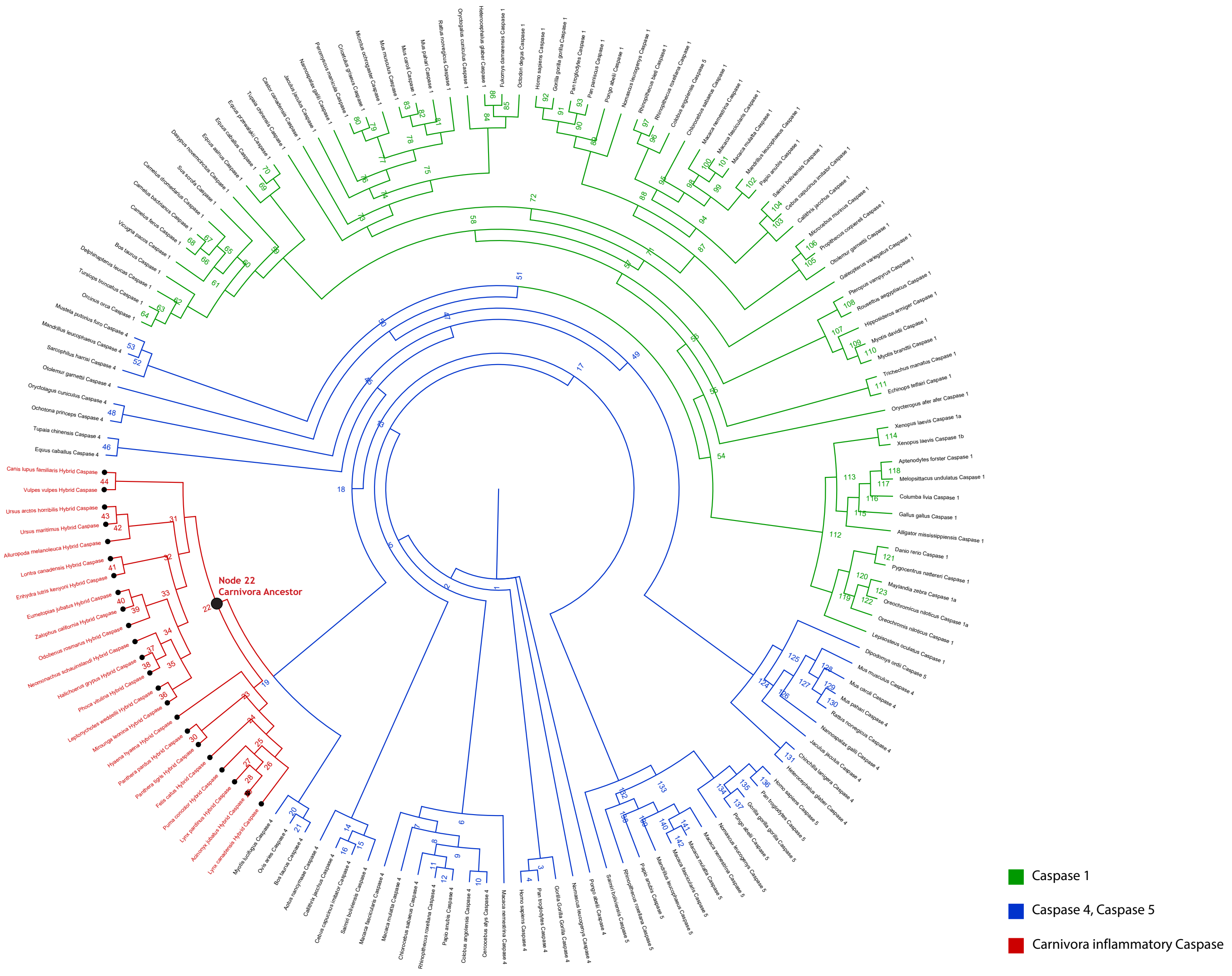

Supplementary Figure 5. Sequence alignment of node 22, human caspases-1 and -4, and the dog inflammatory caspase catalytic domains. Ambiguous residues are represented with asterisks and percent identity values of node 22 to other inflammatory caspases are shown in parenthesis. Catalytic residues His-237 and Cys-285 are indicated in bold letters. Amino acids within loop-341 involved in substrate interaction are underlined. Residue 342 is a major difference within inflammatory caspases and is highlighted in gray. The caspase-1 numbering system is used.

```
hCasp-1       VKLCSLEEAQRIWKQKSAEIYPIMDKSSRTRLALIICNEEFDSIPRRTGAEVDITGMTML
Dog inf casp  LKLCPPETFVKMYKEKAEEIYPIKERKDRTRLALIICNIEFDHLSTRDGAELDIAGMESL
hCasp-4       LKLCPHEEFLRLCKERAEEIYPIKERNNRTRLALIICNTEFDHLPPRNGADFDITGMKEL
Node 22       LKLCPHEEFVKLCKERAEEIYPIKERKDRTRLALIICNTEFDHLPPRNGADLDIAGMKRL
                             * *                                *       *

                                                         237
                                                          |
hCasp-1       LQNLGYSVDVKKNLTASDMTTELEAFAHRPEHKTSDSTFLVFMSHGIREGICGKKHSEQV
Dog inf casp  LEGLGYSVVVKRKLTAKGMESVLREFAARPEHKSSDSTFLVLMSHGILNGICGTAHSVEN
hCasp-4       LEGLDYSVDVEENLTARDMESALRAFATRPEHKSSDSTFLVLMSHGILEGICGTVHDEKK
Node 22       LEGLGYSVDVKEKLTAKDMESVLRAFAARPEHKSSDSTFLVFMSHGILSGICGTTHSPEN
                        *          *                                *  *

                                            285
                                              |
hCasp-1       PDILQLNAIFNMLNTKNCPSLKDKPKVIIIQACRGDSPGVVWFKDSVGVSGNLSLPTTEE
Dog inf casp  PDVLAYDTIFQIFNNRHCLNLKDKPKVIIIQACRGENPGELWVSDSPKASTDSWTHQPLM
hCasp-4       PDVLLYDTIFQIFNNRNCLSLKDKPKVIIVQACRGANRGELWVRDSPASLEVASSQSSEN
Node 22       PDVLPYDTIFQIFNNRNCLSLKDKPKVIIVQACRGENLGELWVSDSPAAPTDSSSQSPEN
                                                               *      * *

                                         342
                                           |
hCasp-1       FEDDAIKKAHIEKDFIAFCSSTPDNVSWRHPTMGSVFIGRLIEHMQEYACSCDVEEIFRK
Dog inf casp  LQSDAIHKVHVEKDFIAFCSSTPHNVSWRHITKGSLFIAQLITCFQKYSWCCHLEGVFRK
hCasp-4       LEEDAVYKTHVEKDFIAFCSSTPHNVSWRDSTMGSIFITQLITCFQKYSWCCHLEEVFRK
Node 22       LEDDAIYKVHVEKDFIAFCSSTPHNVSWRDVTKGSLFITQLITCFQKYSWCCHLEEVFRK
                *   *                       *
                                      341-loop
hCasp-1       VRFSFEQPDGRAQMPTTERVTLTRCFYLFPGH (60%)
Dog inf casp  VQQSFEKPDVKAQMPTIERVSMTRYFYLFPGH (85.7%)
hCasp-4       VQQSFETPRAKAQMPTIERLSMTRYFYLFPGN (84.2%)
Node 22       VQQSFEKPNVKAQMPTIERLSMTRYFYLFPGN
```

Supplementary figure 6. Substrate velocity plots were utilized to calculate k_{cat}/K_M of the dog inflammatory caspase and the reconstructed ancestral caspase of node 22 against a broadly utilized caspase substrate (Ac-WEHD-AFC) and two caspase-1-optimized substrates (Ac-WQPD-ACC and Ac-FEAD-ACC) (Related to table 2).

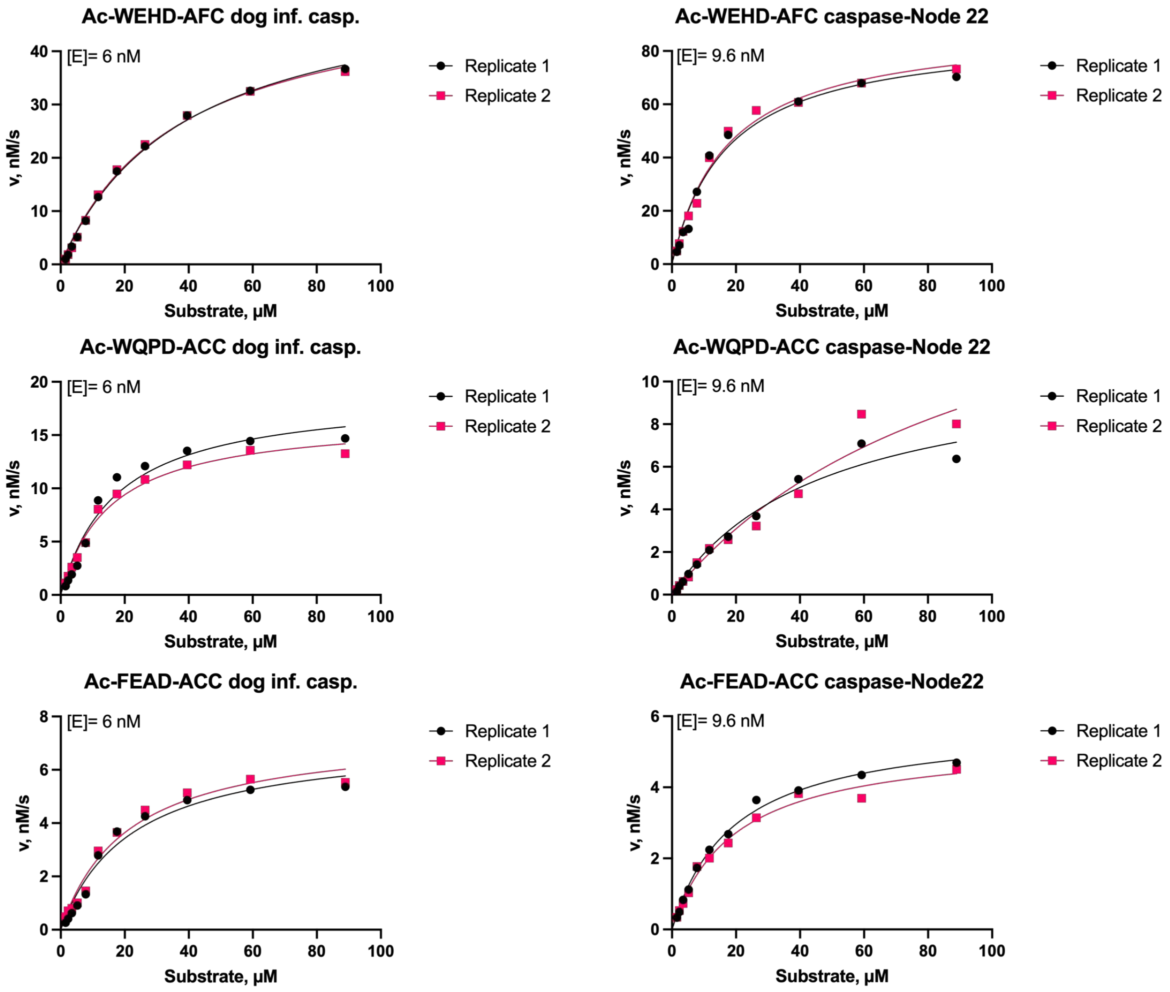

Supplement: Supplemental Tables S1–S2 and Figures S1–S6 [file mmc1.pdf]
